# Methyl-Arginine Profile of Brain from Aged PINK1-KO+A53T-SNCA Mice Suggests Altered Mitochondrial Biogenesis

**DOI:** 10.1155/2016/4686185

**Published:** 2016-03-01

**Authors:** Georg Auburger, Suzana Gispert, Nadine Brehm

**Affiliations:** Experimental Neurology, Goethe University Medical School, 60590 Frankfurt am Main, Germany

## Abstract

Hereditary Parkinson's disease can be triggered by an autosomal dominant overdose of alpha-Synuclein (SNCA) or the autosomal recessive deficiency of PINK1. We recently showed that the combination of PINK1-knockout with overexpression of A53T-SNCA in double mutant (DM) mice potentiates phenotypes and reduces survival. Now we studied brain hemispheres of DM mice at age of 18 months in a hypothesis-free approach, employing a quantitative label-free global proteomic mass spectrometry scan of posttranslational modifications focusing on methyl-arginine. The strongest effects were documented for the adhesion modulator CMAS, the mRNA decapping/deadenylation factor PATL1, and the synaptic plasticity mediator CRTC1/TORC1. In addition, an intriguing effect was observed for the splicing factor PSF/SFPQ, known to interact with the dopaminergic differentiation factor NURR1 as well as with DJ-1, the protein responsible for the autosomal recessive PARK7 variant of PD. CRTC1, PSF, and DJ-1 are modulators of PGC1alpha and of mitochondrial biogenesis. This pathway was further stressed by dysregulations of oxygen sensor EGLN3 and of nuclear TMPO. PSF and TMPO cooperate with dopaminergic differentiation factors LMX1B and NURR1. Further dysregulations concerned PRR18, TRIO, HNRNPA1, DMWD, WAVE1, ILDR2, DBNDD1, and NFM. Thus, we report selective novel endogenous stress responses in brain, which highlight early dysregulations of mitochondrial homeostasis and midbrain vulnerability.

## 1. Introduction

Idiopathic Parkinson's disease (PD) is the second most frequent age-associated neurodegenerative disease. It manifests itself with a movement disorder characterized by hypokinesia, rigidity, rest tremor, and postural instability. The underlying neuron loss exhibits preferential affection of the midbrain dopaminergic neurons. Within the cytoplasm of degenerating neurons, protein aggregates form and coalesce to the so-called Lewy bodies and Lewy neurites, in a process that ascends from olfactory and autonomous neurons via the midbrain to the cerebral cortex [1]. The main component of these inclusion bodies is alpha-Synuclein [2]. This protein plays a key role in the pathogenesis and the transmissibility of PD [3]. Moreover, within the past decades, so many other risk factors have been identified such that now the crucial task of understanding their interactions and shared downstream effects has to be prioritized.

In sporadic PD patients without a positive family history, genome wide investigations of genetic risk factors have identified variants at the genes alpha-Synuclein (*SNCA*) and Tau (*MAPT*) as the main contributors [4]. Alpha-Synuclein is a small lipid-membrane associated protein with chaperone features which is concentrated at presynaptic vesicles [5], but it is also found at the interface between mitochondria and the endoplasmic reticulum [6]. Tau is a microtubule-associated protein that is crucial for axonal organelle transport and growth [7].

Familial PD comprises about 10% of all PD cases [5]. Autosomal dominant forms of PD can be caused by the gain-of-function of alpha-Synuclein through various missense mutations such as A53T (responsible for the PARK1 variant of PD) or through elevated gene dosage (PARK4 variant) [8]. Alpha-Synuclein gain-of-function leads to cumulative mitochondrial damage [9–11], while the absence of alpha-Synuclein renders neurons resistant to mitochondrial stressors [12, 13]. Autosomal recessive forms of PD have been associated very clearly with dysfunctional mitochondria and oxidative stress. A possible cause is (1) the loss-of-function of the mitochondrially targeted ubiquitin kinase PINK1 (responsible for the PARK6 variant) [14, 15], which is known for its role in mitochondrial repair by mRNA translation or fusion [16, 17] and in the autophagic degradation of mitochondria [18]. A possible cause is also (2) the loss-of-function of the PINK1-activated ubiquitin ligase PARKIN (PARK2 variant) [19, 20], which is known as a cytoplasmic regulator of trophic signals [21], but may relocalize to dysfunctional mitochondria and carry out mitophagy [22]. Yet a further cause is (3) the loss-of-function of multifunctional DJ-1 (PARK7 variant), known as an oxidation-sensitive protein that sequestrates the nuclear corepressor PSF, thus regulating the transcriptional regulation of antioxidant defense, DNA repair, and dopamine synthesis [23]. A final cause to be mentioned is (4) the loss-of-function of the lysosomal degradation enzyme Glucocerebrosidase (GBA), which influences the degradation and aggregation of alpha-Synuclein [24, 25].

Given that most PD cases have a polygenic or multifactorial origin, we have recently shown in a digenic mouse modelling approach that the combination of PINK1-KO with overexpression of A53T-SNCA in double mutant (DM) mice potentiates the phenotypes and impairs survival. Lewy-body-like pSer129-SNCA positive aggregates become detectable in the brain tissue after the age of 1 year in these DM mice, and marked mitochondrial mRNA dysregulation and DNA damage marker anomalies were documented, with the spontaneous movements being progressively reduced from the age of 3 months [26].

In view of the prime importance of posttranslational modifications in the regulation of mitophagy and PD [27], we exploited these digenic PD model brains further in several parallel characterization approaches to identify molecular events, which accompany the advent of inclusion bodies and subsequent lethality. The strongest lysine-ubiquitination target observed in brain of the aged DM mice was of course the overexpressed pathogenic alpha-Synuclein [26].

Addressing epigenetics and focusing on the lysine acetylation of proteins, we observed only sparse histone acetylation changes and tubulin acetylation changes, but documented dramatic deficits of mitochondrial acetylation levels at the mouse age of 18 months [28].

Now another hypothesis-free, quantitative label-free global proteomic mass spectrometry scan of posttranslational modifications (PTMscan®) was employed, focusing on mono-methyl-arginine, a crucial modulator of transcription factors and splicing factors [29, 30]. Thus we aimed to complement our existing knowledge about the global transcriptome profile of the DM brain with a pioneer survey of its key regulators. To our knowledge there is no publication so far on the global mono-methyl-arginine profile of brain in a neurodegenerative disorder.

Epigenetic modifications, in particular the methylation of DNA and histones have been characterized in great detail, and for the PD-susceptible midbrain dopaminergic neurons a crucial regulation of PITX3/ADH2/RA/NURR1/SIN3A/PSF through this process was described [31]. In contrast, almost nothing is known about the role of methyl-arginine modifications of other nuclear and cytoplasmic proteins, which have recently been demonstrated to exist [32]. Published reports only provide proof-of-principle that the global methyl-arginine modifications of neural cells depend on trophic cell state [33].

## 2. Materials and Methods

### 2.1. Breeding and Ageing of DM Mice with Homozygosity for* Pink1* ^−/−^ and for A53T-SNCA Overexpression

Our generation, ageing, and characterization of the DM mice were reported before [26]. In brief, the genetic background contains 129/SvEv and FVB/N in a 50 : 50 distribution on average, similar to the WT control mice that were aged F1-hybrids from a crossbreeding of 129/SvEv and FVB/N mice descended from littermates of the respective single mutant animals. The mice were kept in individually ventilated cages under 12 h light cycle with food and water* ad libitum*. Sentinel mice and regular health monitoring including blood tests for viral and parasite infections uncovered no pathology. Housing of animals was in accordance with the German Animal Welfare Act, the Council Directive of 24 November 1986 (86/609/EWG) with Annex II and the ETS123 (European Convention for the Protection of Vertebrate Animals). The mice under investigation were bred and aged at the FELASA-certified Central Animal Facility (ZFE) of the Frankfurt University Medical School. After decapitation, the organs were removed and immediately frozen in liquid nitrogen.

### 2.2. Global Mono-Methyl-Arginine Motif Survey by Label-Free Mass Spectrometry

Brain hemispheres from mice at age of 18 months (three DM versus three WT matched for male sex) were dissected in parallel, snap-frozen in liquid nitrogen, stored at −80°C, and shipped on dry ice for the commercial MethylScan® procedure by Cell Signaling Technology, Inc. [34, 35]. In short, tissue extracts were protease-digested and subjected to C18 solid-phase extraction. The lyophilized peptides were immunoprecipitated by protein-A/G-agarose-immobilized mono-methyl-arginine motif antibodies #8015/8711. Peptides were loaded directly onto a 10 cm × 75 *μ*m PicoFrit capillary column packed with Magic C18 AQ reversed-phase resin. The column was developed with a 90 min linear gradient of acetonitrile in 0.125% formic acid delivered at 280 nL/min. The MS parameter settings were as follows: MS Run Time 96 min, MS1 Scan Range (300.0–1500.00), and Top 20 MS/MS (Min Signal 500, Isolation Width 2.0, Normalized Coll. Energy 35.0, Activation-Q 0.250, Activation Time 20.0, Lock Mass 371.101237, Charge State Rejection Enabled, Charge State 1+ Rejected, Dynamic Exclusion Enabled, Repeat Count 1, Repeat Duration 35.0, Exclusion List Size 500, Exclusion Duration 40.0, Exclusion Mass Width Relative to Mass, Exclusion Mass Width 10 ppm). MS/MS spectra were evaluated using SEQUEST 3G and the Sorcerer 2 platform from Sage-N Research (v4.0, Milpitas, CA, USA) [36]. Searches were performed against the most recent update of the NCBI* Mus musculus* database with mass accuracy of ±50 ppm for precursor ions and 1 Da for product ions. The results were filtered with mass accuracy of ±5 ppm on precursor ions and presence of the intended motif (Me-R). The peptide identification with relative quantification by mass spectrometry (MS) occurred by LC-MS/MS analysis using LTQ-Orbitrap-VELOS with ESI-CID Sorcerer search.

With double injections of the 6 biological samples, 12 LC-MS/MS experiments were conducted and bioinformatically processed, using the maximum % coefficient of variation (% CV) to control replicate reproducibility. Using a 5% default false positive rate to filter the Sorcerer results, this procedure yielded a total of 2,218 redundant methylated peptide assignments to 971 nonredundant ubiquitinated peptides. The quantitative data from the three control WT mice were averaged to compare each DM mouse individually and derive the respective fold change. The original data are available from the authors upon request.

## 3. Results

**Figure 1:**
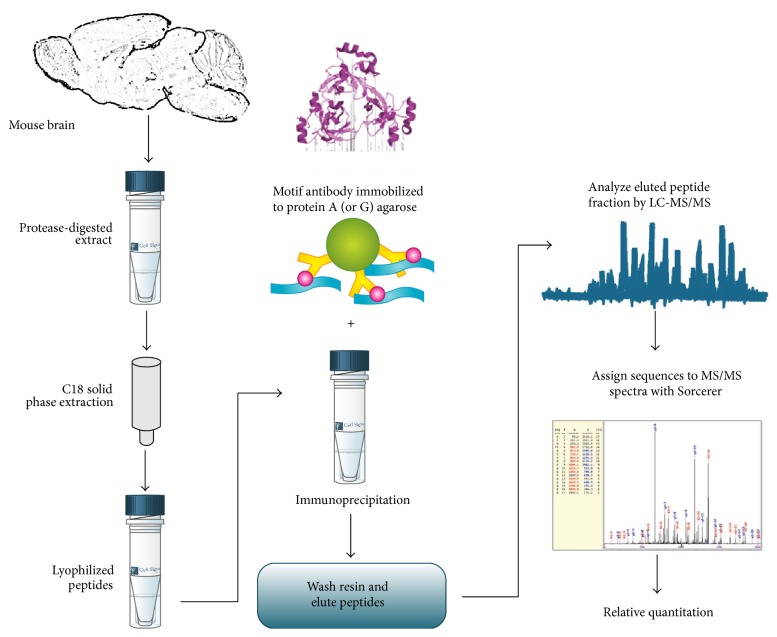
Workflow chart illustrating the technical approach to quantify the mono-methyl-arginine-modification of peptides throughout the global brain proteome in a quantitative and label-free manner by immunoprecipitation and mass spectrometry. The immunoprecipitation step illustrates the motif antibody (above), the agarose beads (green circle), its immunoglobulin coating (yellow), and the binding of digested peptides (blue) with mono-methyl-arginine modifications (pink). Graphic elements from internet-sites (http://www.cellsignal.com/common/content/content.jsp?id=proteomics-discovery and http://media.cellsignal.com/www/pdfs/proteomics/methylscan_workflow.pdf) were used with permission of Cell Signaling Inc.

**Figure 2:**
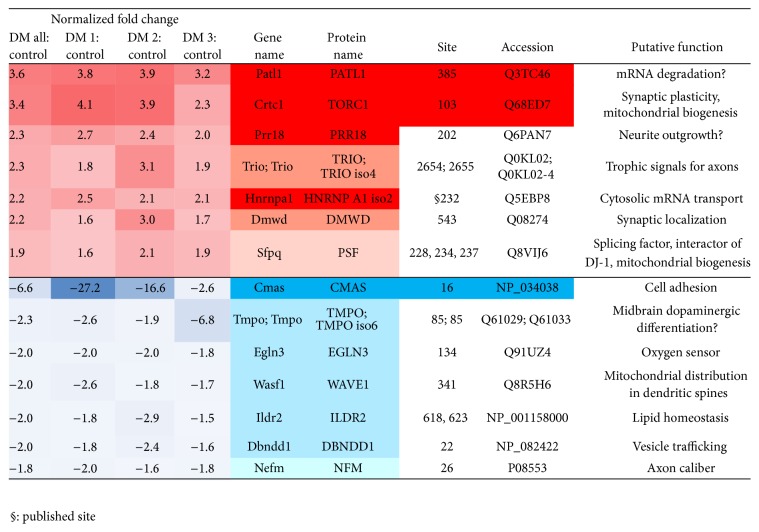
Mouse brain; trypsin digest; mono-methyl-arginine motif antibody #8015/8711. JW Goethe University Hospital (Q153802_8_25) MethylScan results.

The global brain proteome of three 18-month-old DM mice versus three matched wildtype (WT) mice was analyzed in a quantitative label-free mass spectrometry approach (see Figure 1) for the abundance of mono-methyl-arginine (Me-R) motifs (MethylScan). The original data were filtered for consistency and effect size. We excluded factors where each of the three DM mice did not show the same direction of change. We also excluded changes smaller than 1.5-fold. The remaining observations comprised only 7 upregulation effects and only 7 downregulation effects, which are shown in Figure 2, ordered by effect size (illustrating upregulations in red and downregulations in blue, highlighting relative effect sizes of different animals with a heat map color scale and emphasizing proteins with consistent >2-fold changes by a more intense coloring).

### 3.1. Upregulations in Brains from Aged DM Mice

Me-R385-PATL1 (protein PAT1 homolog 1) showed a 3.6-fold change in brains from aged DM mice.

Me-R103-CRTC1/TORC1 (CREB regulated transcription activator or transducer of regulated CAMP response element-binding protein) showed a 3.4-fold change.

Me-R202-PRR18 (proline-rich region 18) showed a 2.3-fold change.

Me-R2654-TRIO (triple functional domain Rho Guanine Nucleotide Exchange Factor) and Me-R2655-TRIO isoform 4 showed a 2.3-fold change.

Me-R232-HNRNPA1 isoform 2 (heterogeneous nuclear ribonucleoprotein A1) showed a 2.2-fold change.

Me-R543-DMWD (dystrophia myotonica WD repeat-containing protein) showed a 2.2-fold change.

Me-R228-PSF/SFPQ (polypyrimidine tract-binding protein-associated-splicing factor or splicing factor and proline- and glutamine-rich), Me-R234-PSF/SFPQ, and Me-R543-PSF/SFPQ showed a 1.9-fold change.

### 3.2. Downregulations in Brains from Aged DM Mice

Me-R16-CMAS (N-acetylneuraminate cytidylyltransferase) showed a −6.6-fold change.

Me-R85-TMPO (thymopoietin- or lamina-associated polypeptide 2) showed a −2.3-fold change.

Me-R134-EGLN3 (Egl nine homolog 3 or prolyl hydroxylase domain-containing protein 3, PHD3) showed a −2.0-fold change.

Me-R341-WAVE1 (Wiskott-Aldrich syndrome protein family member 1) showed a −2.0-fold change.

Me-R618-ILDR2 (immunoglobulin-like domain-containing receptor 2) and Me-R623-ILDR2 showed a −2.0-fold change.

Me-R22-DBNDD1 (dysbindin domain-containing protein 1) showed a −2.0-fold change.

Me-R26-NFM (neurofilament medium peptide) showed a −1.8-fold change.

**Figure 3:**
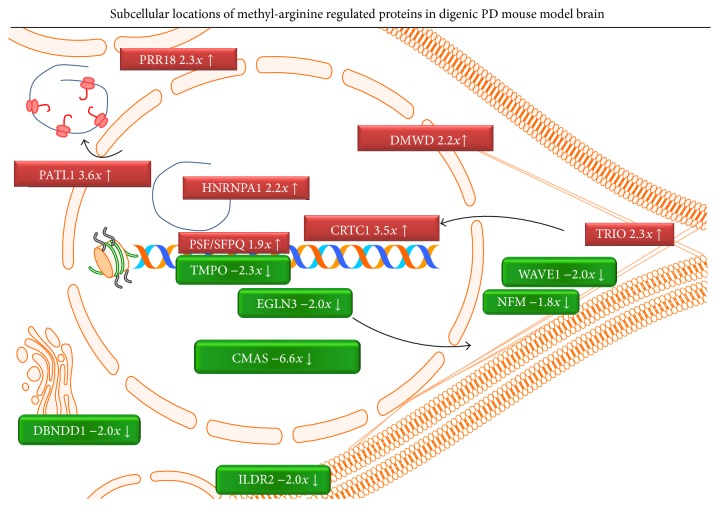
Subcellular localization scheme of proteins with mono-methyl-arginine that show changed abundance. The locations are shown according to GeneCards database information and published literature; diagrams from Motifolio toolkits were used for drawing. Red boxes represent upregulation events; green ellipses were used for downregulations, in sizes proportional to fold changes. Apart from the nuclear envelope around DNA and a histone in the center of the picture, the rough endoplasmic reticulum with associated translating RNA is shown in the upper left corner, the Golgi apparatus on the left side, and the smooth endoplasmic reticulum in the lower left corner; cytoskeletal elements extend from the right side of the nucleus to the cell membranes, which form a neurite towards the right side and the plasma membrane of an adjacent cell is shown in the lower right corner. Arrows indicate nuclear export and import.

Both the upregulation and the downregulation events clustered among proteins with nuclear localization, shuttling to cytoplasmic and cytoskeletal positions, as illustrated in Figure 3. Dysregulations were not observed for chromatin binding factors, for G protein regulators, for vesicle proteins, for translation factors, for membrane receptors/channels/transporters, and for membrane adaptors/scaffolds, which are known to undergo methyl-arginine modifications [35].

## 4. Discussion

This study of the global mono-methyl-arginine profile in brain hemispheres by quantitative label-free mass spectrometry is the first of its kind in a neurodegenerative disorder. Although this approach might be expected to reveal epigenetic anomalies, consistent strong histone methylation changes were not observed. Of course, any global survey may produce false positive and false negative errors, but this screening yielded a surprisingly high enrichment of factors that were previously connected to neurodegeneration, PD, or dopaminergic differentiation. Furthermore, a considerable number of the identified factors are interactors in protein complexes, suggesting that they constitute particularly promising candidates for follow-up experiments. Below we comment on the relevance of each factor individually.

Regarding the upregulated events, little is known about PATL1, but it is enriched at splicing speckles and shuttles between nucleus and cytoplasm. It was observed that a viral infection may disrupt PATL1-localization at P-bodies as the sites of mRNA degradation and sequestrate PATL1 to the vicinity of lipid droplets [37]. This may be relevant given that the PINK1/PARKIN pathway was shown to mediate the cellular resistance to infections [38, 39].

CRTC1/TORC1 senses the convergence of calcium/cAMP/phosphorylation signals, relocalizes from the synapse to the nucleus in an activity-dependent manner, and triggers transcriptional responses that are key to the late phase of long-term potentiation and synaptic plasticity [39–41]. This observation is very intriguing, given that impaired synaptic function and plasticity in the nigrostriatal and corticostriatal brain projections have already been demonstrated for these mice due to their A53T-SNCA overexpression [42–52]. CRTC1 is also a potent coactivator of PGC1alpha and inducer of mitochondrial biogenesis that modulates the growth of neurites [53, 54]. CRTC1 has been implicated in several neurodegenerative diseases already. Synaptic activity induces CRTC1 dephosphorylation (Ser151), nuclear translocation, and CRTC1-dependent transcription in the hippocampus, which is deficient in Alzheimer's disease models. CRTC1 overexpression reverses amyloid-beta-induced spatial learning and memory deficits [55–57]. In models of Huntington's disease, mutant huntingtin protein interferes with the TORC1-CREB interaction to repress transcription of brain-derived neurotrophic factor [58], and also the depletion of CRTC1 contributes to Huntington's disease [59]. Moreover, CRTC1 phosphorylation is crucial for the outcome after cerebral ischemia [60]. Thus, the increased methylation at R103-CRTC1 in our PD model confirms an important role of this DNA-binding protein in neurodegenerative processes and identifies a novel regulation mechanism.

No functional insights exist on PRR18, which has predicted localizations in the nucleus and endoplasmic reticulum, being coexpressed with neurite outgrowth regulators such as Lingo1 (leucine-rich repeat and Ig domain-containing 1) in mouse brain according to the STRING Heidelberg protein interaction database.

TRIO controls the directional extension of axons [61], modifying the signaling by FGFR and GPCR pathways and acting through AKT signaling to influence mitochondrial apoptosis [62]. Its DBL/GEF domains are thought to influence the production of membrane ruffles and the formation of stress fibers.

HNRNPA1 is involved in the packaging of pre-mRNA into hnRNP particles, the transport of poly A+ mRNA from the nucleus to the cytoplasm, and the selection of splice sites. It is coregulated together with the splice factor PSF/SFPQ (see below) by stress-induced phosphorylation signals [63]. Mutations of HNRNPA1 were reported in the motoneuron degeneration disorders Amyotrophic Lateral Sclerosis (ALS) and Frontotemporal Lobar Degeneration (FTLD) [64].

Although little functional insight exists on DMWD, it is highly abundant in neurons and concentrated in synaptic connections [65]. It contains multiple WD40-repeats, which have been implicated in cytoskeleton assembly, pre-mRNA processing, and signal transduction. In the skeletal muscle degeneration disorder named myotonic dystrophy, the DMWD levels were found deficient [66].

In spite of the modest effect size, the upregulation of PSF/SFPQ methylation is intriguing because PSF/SFPQ in the nucleus interacts directly with the protein DJ-1, which is responsible for autosomal recessive juvenile Parkinson's disease [67]. DJ-1 inhibits the sumoylation of PSF/SFPQ, while elevating the expression of the dopamine homeostasis factors tyrosine hydroxylase (TH) and vesicular monoamine transporter 2 (VMAT2) [68, 69] as well as modulating the levels of PGC1alpha as a key factor of mitochondrial biogenesis [70], which is also regulated by CRTC1/TORC1 above. Nuclear PSF/SFPQ also interacts with FUS (fused in sarcoma), a protein responsible for the motoneuron degeneration disorders ALS and FTLD [71]. Nuclear PSF/SFPQ is recruited to sites of DNA damage [72]. PSF/SFPQ was found to interact directly with the internal ribosomal entry site (IRES) of the DNA repair factor TP53 (p53) [73] and with cytoplasmic PARKIN, which is responsible for the PARK2 variant of autosomal recessive juvenile Parkinson's disease [74]. The observation of its Me-R changes is also intriguing because PSF/SFPQ exists in a nuclear protein complex with LMX1B/PITX3/NR4A2 = NURR1, key factors in the development of midbrain dopaminergic neurons [75, 76], similar to TMPO below. Arginine methylation of PSF/SFPQ by the arginine N-methyltransferase PRMT1 was observed previously and shown to enhance the association with mRNA in mRNP complexes in mammalian cells [77]. The presence of PSF/SFPQ in neuronal RNA transport granules was reported, and its interaction with JNK-kinase depends on stimulation by NGF (Nerve Growth Factor) [78]. An overall role of PSF/SFPQ mRNA effects consisted, for example, in the inhibition of IGF-1-stimulated transcriptional activity and thus the trophic modulation of cells [79]. Like CRTC1, PSF/SFPQ also has been implicated in several neurodegenerative diseases already. SFPQ modulates the splicing of Tau, while Tau mediated the nuclear depletion of PSF/SFPQ in Alzheimer's and Pick's disease together with a cytoplasmic accumulation [80–83], as well as a depletion in the brain of Down syndrome cases [84]. The PSF/SFPQ transcript was upregulated in Alzheimer's disease brain [85]. PSF/SFPQ mislocalized from the neuronal nucleus to the cytoplasm in the motor neuron diseases ALS and FTLD, which were caused by TDP-43 mutations [86, 87]. Thus, also the observation of increased methylation of PSF/SFPQ at Me-R228, Me-R234, and Me-R543 in our PD model supports the relevance of this RNA-binding factor for neurodegenerative processes and describes a new regulation mechanism.

Regarding the downregulated events, the nuclear protein CMAS activates the sugar NeuNAc to the compound CMP-NeuNAc, which is needed for the addition of sialic acid to modulate cell surface glycoprotein and glycolipid interaction, thus modulating cell adhesion. No evidence existed so far that implicated this factor in neurodegenerative disorders, but it seems to be involved in Fragile-X mental retardation [88].

The nuclear protein TMPO interacts with LMX1B and NURR1, two key factors in the development of midbrain dopaminergic neurons [75], similar to PSF/SFPQ above. It has been implicated in dilated cardiomyopathy and in diabetes mellitus type 1 [89, 90], but not in neurodegenerative disorders so far.

The nucleus-cytoplasm shuttling protein EGLN3 acts as cellular oxygen sensor that hydroxylates HIF1A and HIF2A, thus regulating neuronal apoptosis [91]. It is coinduced with TP53-activation in the DNA damage response pathway [92]. Interestingly,* EGLN3* showed downregulated transcript levels in the midbrain-derived dopaminergic neuronal cell line MN9D after treatment with the Parkinsonian neurotoxin MPP+ [93]. These features are quite similar to DJ-1, which acts as an oxygen sensor, regulates HIF1A and TP53, and rescues the MPP+ toxicity of PINK1-deficient dopaminergic neurons [94–98].

WAVE1 acts downstream of Nerve Growth Factor and of the RAC1 GTPase to regulate actin filament reorganization and axonal filopodia formation via its interaction with the Arp2/3 complex [99] and controls dendritic spine morphology and neural activity-induced mitochondrial distribution in dendritic spines [100, 101].* WAVE1* transcription is negatively regulated by the amyloid precursor protein intracellular domain, and WAVE1 protein depletion dramatically reduces amyloid beta levels and restores memory deficits in a mouse model of Alzheimer's disease [102]. WAVE1 coaggregates with hyperphosphorylated Tau and is found in neurofibrillary tangles and abnormal neurites of Alzheimer's disease brain [103].

ILDR2 localizes at tricellular tight junctions, while modulating lipid homeostasis and endoplasmic reticulum stress pathways [104, 105]. Although nothing is known yet about disease associations of ILDR2, mutations in its homologue ILDR1 were shown to be responsible for a neurosensory degeneration disorder resulting in the autosomal recessive hearing impairment DFNB42 [106].

No functional insights exist on DBNDD1. Its homolog dysbindin-1 (DTNBP1 or BLOC1S8) is a component of the BLOC-1 complex, which targets membrane protein cargos into vesicles for delivery into nerve terminals and is thus involved in neurite extension as well as synaptic vesicle trafficking [107]. DTNBP1 regulates the cell surface presence of the dopamine receptor DRD2 and modulates prefrontal activity via the dopamine D2 pathway [108]. A disease association for DBNDD1 is not identified yet, but mutations in DTNBP1 are responsible for the Hermansky-Pudlak syndrome 7 [109], which is characterized by oculocutaneous albinism, prolonged bleeding, and pulmonary fibrosis.

NFM is important for neuronal axon caliber [110]. It is a component of Lewy bodies in Parkinson's disease brains [111] and a reduction of phospho-NFM levels was already observed in PINK1-KO mouse brains [112]. Autoantibodies against NFM are observed in the cerebrospinal fluid and blood serum of individuals with the motoneuron degeneration ALS [113].

For each of these factors, firstly the discovery that they are regulated by methylation and secondly the identity of the specific arginine, both are providing valuable insights. A downside of this novel approach, however, is the fact that no site-specific antibodies are available at present to validate these findings by a technically independent approach. This is a severe limitation of our study. Thus, we can only report in a descriptive manner that the survey supports the relevance of the proteins listed above in the early stage of Parkinsonian neurodegeneration concomitant with the appearance of Lewy-body-like pSer129-SNCA positive protein aggregates and the manifestation of motor deficits in the DM mouse line.

Even if the microscopic detection of pSer129-SNCA positive protein aggregates in the brain becomes possible only in the second year of life of our DM mice, at a submicroscopic level an insidiously progressive pathology might be ongoing much earlier. Alpha-Synuclein would adopt pathological conformations, oligomerize, undergo fibrillation, sequestrate interactor molecules into insolubility, and be compensated by degradation and extrusion efforts, before this process becomes visible in microscopes. In this light it is interesting to note that several proteins that are known to coaggregate with the disease protein in neurodegenerative conditions, did indeed show dysregulated arginine methylation in this screening, namely, NFM (coaggregating with SNCA), WAVE1 (coaggregating with Tau), and PSF/SFPQ (mislocalized from nucleus to cytoplasm by Tau and TDP-43).

It is also interesting to note that no loss of dopaminergic midbrain neurons could be substantiated in the aged DM mice; however, the dysregulated arginine methylation of PSF/SFPQ and TMPO, both of which interact with LMX1B/NURR1 in the regulation of dopaminergic midbrain neuron differentiation and regeneration, suggests that molecular anomalies in these neurons are occurring in a selective and prominent manner, while the neuronal morphology is still intact.

As an additional approach to evaluate the credibility of this survey, we questioned whether the previous transcriptome profile in the brain of aged single mutant A53T-SNCA overexpressing mice or of aged DM mice can be correlated to the dysregulated methylation of transcription factors.

In the case of aged A53T-SNCA mice, the global transcriptome was previously documented by us in the striatal region and dysregulations of a CREB regulated transcription factor named* Atf2* (cyclic AMP-responsive element-binding protein 2) and its upstream regulators* Cnr1* and* Homer1* were among the main observations [44] (see Table S2 of that reference). The same pathway is reflected in the present MethylScan by the CREB regulated transcription factor CRTC1/TORC. This pathway is crucial for trophic signaling, neurite extension, synaptic plasticity and adhesion, processes that are actively regulated by CRTC1/TORC1, TRIO, PSF/SFPQ, CMAS, WAVE1, ILDR2, NFM, and perhaps by PRR18 and DBNDD1 as MethylScan candidates, as well as by PARKIN and DJ-1 as additional causes of autosomal recessive PD [114–117].

In the case of the aged DM mice, the global transcriptome throughout brain hemispheres was previously documented by us to comprise dysregulations of the SNCA-abundance marker and cell adhesion factor* Lect1* (Leukocyte-expressed chemotaxin-1 or chondromodulin-1), of the autophagy factor* Dapk1* (death-associated protein kinase 1) and of the DNA damage marker* H2afx* (H2A histone family, member X) [26].* Lect1* transcription upon demethylation of its core promoter region [118] occurs upon Nerve Growth Factor treatment in a TP53-dependent manner [119], in parallel to converse changes in the levels of HIF-1alpha (Hypoxia-inducible factor 1, alpha subunit) [120].* Dapk1* transcripts are produced in dependence on its promoter methylation which is regulated by the transcription factor TP53. The* Dapk1* transcripts undergo alternative splicing [121].* H2afx* transcript levels and protein localization depend on histone methylation and also on the DNA repair activator TP53 [122, 123]. Clearly the dependence of these three transcripts on the TP53 pathway is reflected in our MethylScan now by the PSF/SFPQ modulation of the TP53-IRES and by the TP53-effects of the PSF/SFPQ-interactor protein DJ1 [96, 124]. It was already observed in the neurodegenerative process of Huntington's disease that H2AFX, ATM, and TP53 are coactivated before the microscopic appearance of aggregates [125] and that there is a relative deficit of TP53/H2AFX dependent DNA repair [126]. Again, this pathway is also modulated by PARKIN and DJ-1 as additional causes of autosomal recessive PD [96, 97, 117, 127–133].

TP53 via PARKIN was observed to modulate glucose metabolism and the Warburg effect [132], mitochondrial length [134], and mitophagy [129, 130]. Indeed, the mitochondrial biogenesis pathway also could be affected in the brains of the aged DM mice according to the MethylScan findings, given that CRTC1/TORC1 and PSF/SFPQ in interaction with DJ-1 are known modulators of PGC1alpha, the central inducer of mitochondrial biogenesis [135]. These arginine methylations could represent a cellular compensation effort. Given that the A53T-SNCA overexpression in the DM mice is known to exert mitochondrial toxicity but that dysfunctional mitochondria cannot be eliminated through selective mitophagy in the DM mice due to the absence of PINK1, one would expect dysfunctional mitochondria to accumulate in neurons in a similar manner as they accumulate as ragged red fibers in muscles of MERRF patients. However, such a neuronal accumulation of dysfunctional mitochondria could not be observed by microscopy or by immunoblot assessments of mitochondrial mass in the DM mice. Thus, a compensatory downregulation of mitochondrial biogenesis would appear to be a logical explanation. Furthermore, the altered methylation of the oxygen sensor EGNL3 may also represent an adaptive cellular response to the increasing mitochondrial dysfunction and oxidative stress in the brain of aged DM mice.

Of course it is interesting now to speculate how the mitochondrial dysfunction is perceived and how it elicits the compensatory efforts and downstream pathology that we have documented. It has been shown in midbrain dopaminergic neurons that neuronal activity-dependent calcium entry through L-type calcium channels triggers oxidative stress and promotes alpha-Synuclein aggregation, while the effect of calcium on oxidative stress is potentiated by the formation of alpha-Synuclein Lewy-body-like aggregates [41, 55].

Mitochondrial dysfunction induces PINK1 expression in a calcium-dependent manner [56], while PINK1 depletion compromises calcium homeostasis [3]. Both alpha-Synuclein and the PINK1 downstream effector PARKIN were shown to act at contact zones between mitochondrial membranes and endoplasmic reticulum, where calcium homeostasis and mitochondrial dynamics are controlled [1, 16, 17]. CRTC1/TORC1 depends on neuronal activity-dependent calcium in its translocation to the nucleus, where it acts to modulate mitochondrial homeostasis [18, 40, 53]. Thus, alpha-Synuclein triggered toxicity and PINK1 deficiency have convergent effects on calcium homeostasis, which may be sensed by CRTC1 and elicit compensatory efforts of mitochondrial biogenesis.

## 5. Conclusion

This pioneer study of the global mono-methyl-arginine profile of brain in a neurodegeneration mouse model is reporting a small number of novel posttranslational modifications with substantial fold changes. These alterations occur mostly in nuclear factors previously implicated in other neurodegenerative diseases and are clustering in the pathways of dopaminergic neuron differentiation and of mitochondrial biogenesis and antioxidant protection. Although an independent validation with other techniques is not possible and our study thus is severely limited, the data fit well with previous transcriptome findings and with functional changes of long-term-depression previously documented to be triggered by the alpha-Synuclein mutation in these mice. Particularly interesting is the increased methylation of the synaptic plasticity modulator CRTC1. We speculate (1) that the CRTC1 changes are responding to altered calcium homeostasis and represent a compensatory effort to modulate mitochondrial biogenesis and (2) that they are due to the impaired mitochondrial autophagy in these mice. Thus, this methyl-arginine profiling effort of digenic PD mouse models identifies dysregulations of CRTC1 as a potential key factor, where the effects of alpha-Synuclein on synaptic plasticity converge with the effects of PINK1 on mitochondrial quality control.
